# Loss of Y in leukocytes as a risk factor for critical COVID-19 in men

**DOI:** 10.1186/s13073-022-01144-5

**Published:** 2022-12-14

**Authors:** Bożena Bruhn-Olszewska, Hanna Davies, Daniil Sarkisyan, Ulana Juhas, Edyta Rychlicka-Buniowska, Magdalena Wójcik, Monika Horbacz, Marcin Jąkalski, Paweł Olszewski, Jakub O. Westholm, Agata Smialowska, Karol Wierzba, Åsa Torinsson Naluai, Niklas Jern, Lars-Magnus Andersson, Josef D. Järhult, Natalia Filipowicz, Eva Tiensuu Janson, Sten Rubertsson, Miklós Lipcsey, Magnus Gisslén, Michael Hultström, Robert Frithiof, Jan P. Dumanski

**Affiliations:** 1 Department of Immunology, Genetics and Pathology and Science for Life Laboratory, Uppsala University, Uppsala, Sweden; 2 3P-Medicine Laboratory, Medical University of Gdańsk, Dębinki 7, 80-211 Gdańsk, Poland; 3 National Bioinformatics Infrastructure Sweden, Department of Biochemistry and Biophysics, Stockholm University, Science for Life Laboratory, Stockholm, Sweden; 4 Department and Clinic of Rheumatology, Clinical Immunology, Geriatrics and Internal Medicine, Medical University of Gdańsk, Gdańsk, Poland; 5 Department of Laboratory Medicine, Institute of Biomedicine and Biobank Core Facility, Sahlgrenska Academy at the University of Gothenburg, Gothenburg, Sweden; 6 Department of Infectious Diseases, Institute of Biomedicine, Sahlgrenska Academy, University of Gothenburg, Gothenburg, Sweden; 7 Department of Infectious Diseases, Region Västra Götaland, Sahlgrenska University Hospital, Gothenburg, Sweden; 8 Zoonosis Science Center, Department of Medical Sciences, Uppsala, Sweden, Uppsala University, Uppsala, Sweden; 9 Department of Medical Sciences, Endocrine Oncology Unit, Uppsala University, Uppsala, Sweden; 10 Department of Surgical Sciences, Anesthesiology and Intensive Care, Uppsala University, Uppsala, Sweden; 11 Hedenstierna laboratory, Department of Surgical Sciences, Uppsala University, Uppsala, Sweden; 12 Integrative Physiology, Department of Medical Cell Biology, Uppsala University, Uppsala, Sweden

## Abstract

**Background:**

The COVID-19 pandemic, which has a prominent social and economic impact worldwide, shows a largely unexplained male bias for the severity and mortality of the disease. Loss of chromosome Y (LOY) is a risk factor candidate in COVID-19 due to its prior association with many chronic age-related diseases, and its impact on immune gene transcription.

**Methods:**

Publicly available scRNA-seq data of PBMC samples derived from male patients critically ill with COVID-19 were reanalyzed, and LOY status was added to the annotated cells. We further studied LOY in whole blood for 211 COVID-19 patients treated at intensive care units (ICU) from the first and second waves of the pandemic. Of these, 139 patients were subject to cell sorting for LOY analysis in granulocytes, low-density neutrophils (LDNs), monocytes, and PBMCs.

**Results:**

Reanalysis of available scRNA-seq data revealed LDNs and monocytes as the cell types most affected by LOY. Subsequently, DNA analysis indicated that 46%, 32%, and 29% of critically ill patients showed LOY above 5% cut-off in LDNs, granulocytes, and monocytes, respectively. Hence, the myeloid lineage that is crucial for the development of severe COVID-19 phenotype is affected by LOY. Moreover, LOY correlated with increasing WHO score (median difference 1.59%, 95% HDI 0.46% to 2.71%, *p*=0.025), death during ICU treatment (median difference 1.46%, 95% HDI 0.47% to 2.43%, *p*=0.0036), and history of vessel disease (median difference 2.16%, 95% HDI 0.74% to 3.7%, *p*=0.004), among other variables. In 16 recovered patients, sampled during ICU stay and 93–143 days later, LOY decreased significantly in whole blood and PBMCs. Furthermore, the number of LDNs at the recovery stage decreased dramatically (median difference 76.4 per 10,000 cell sorting events, 95% HDI 55.5 to 104, *p*=6e−11).

**Conclusions:**

We present a link between LOY and an acute, life-threatening infectious disease. Furthermore, this study highlights LOY as the most prominent clonal mutation affecting the myeloid cell lineage during emergency myelopoiesis. The correlation between LOY level and COVID-19 severity might suggest that this mutation affects the functions of monocytes and neutrophils, which could have consequences for male innate immunity.

**Supplementary Information:**

The online version contains supplementary material available at 10.1186/s13073-022-01144-5.

## Background

Leukocytes from aging males frequently show mosaic loss of chromosome Y (LOY) [1, 2]. It is detectable in whole blood DNA from >40% of men above the age of 70 years [3] and reaches 57% in the analysis of 93-year-old men, indicating an increasing frequency with age [4]. Recent single-cell analyses of peripheral blood mononuclear cells (PBMCs) from 29 aging men (median age 80 years, range 64–94 years) identified cells with LOY in every studied subject, making this the most common post-zygotic mutation [5]. LOY is most common in leukocytes, but has also been detected in other tissues although with considerably lower frequencies [4, 6]. Notably, the results of the serial analysis of male blood samples showed that LOY is a dynamic process [7]. Known risk factors for LOY include age, smoking, and germline predisposition [1–3, 8]. LOY likely causes a clonal expansion of affected cells and may coexist with mutations causing clonal hematopoiesis of indeterminate potential (CHIP) [9].

LOY in DNA from whole blood has been associated with increased risk for all-cause mortality and many chronic age-related diseases such as hematological and non-hematological cancers, Alzheimer’s disease, diabetes, and cardiovascular events, among others [1, 2, 6, 10–15]. Thus, carriers of LOY in leukocytes have an increased risk for diseases both inside and outside of the hematopoietic system, and the mechanism(s) behind these associations largely remain to be elucidated. Recent studies suggest that LOY affects different lineages of hematopoietic cells with dissimilar frequencies. LOY could have a direct physiological role through *L*OY-*A*ssociated *T*ranscriptional *E*ffects (LATE) on global gene expression in a pleiotropic manner, as well as being a biomarker of genomic instability in somatic tissues [5]. Moreover, dysregulation of immune genes, including pathways related to viral life-cycle, was pronounced in LOY cells [5, 16].

Coronavirus disease 2019 (COVID-19) is caused by the new SARS-CoV-2 virus [17]. Critically affected patients present with bilateral pneumonia, in many cases, progressing to acute respiratory distress syndrome with a strong decrease in pulmonary function requiring treatment with non-invasive or invasive mechanical ventilation [18]. Results suggest that neutrophil activation is a hallmark of COVID-19 together with lymphocytopenia and that patients with the critical disease show substantial elevation of circulating neutrophils [19–21]. In COVID-19 patients, neutrophils adopt a so-called low-density phenotype, when they are activated and prone to spontaneous release of neutrophil extracellular traps (NETs) in capillaries. Intra-vascular aggregations of NETs lead to occlusion of the affected vessels, disturbed microcirculation, and organ damage. Furthermore, hyper-coagulation and thromboembolic events are also contributing to the mortality from COVID-19 [22, 23]. These symptoms are possibly a consequence of dysfunction in myeloid cell lineages and growing evidence corroborates this hypothesis [24, 25]. Another feature of the disease is a strong and largely unexplained bias of males who are critically ill and die from COVID-19. Of 7874 patients treated and registered since March 2020 at Intensive Care Units (ICU) in Sweden, 70.1% were men [26]. Other studies of COVID-19 patients confirm this strong male bias [27, 28] and this aspect of COVID-19 pathogenesis is understudied [29]. Chromosome X-linked recessive *TLR7* gene deficiency explains about 1% of life-threatening COVID-19 in males below 60 years of age [30]. Moreover, pre-existing neutralizing auto-antibodies against type I IFN in about 10% of male COVID-19 patients have been linked to life-threatening diseases [31]. However, there might exist an additional, so far unidentified male-specific risk factor, predisposing men for a life-threatening disease course of COVID-19 and LOY could be a suitable candidate for such a factor.

We hypothesized that LOY in cells from the myeloid lineage might be a male-specific risk factor linked to the development of critical COVID-19 disease. We concentrated our study on 211 critically ill males that were treated at ICUs in Sweden. COVID-19 patients treated at ICUs represent a suitable cohort for genotype-phenotype association studies since their records of medical treatment are comprehensive and this subset of patients show a considerable inter-individual clinical variability in the course of COVID-19 disease (WHO score 6-10).

## Methods

### Study design, sample collection, and clinical variables

Male patients with COVID-19 were enrolled in Sweden, at Uppsala University Hospital (as part of the PronMed-study) and Sahlgrenska University Hospital in Gothenburg with confirmed positive SARS-CoV-2 nasopharyngeal test verified by RT-PCR. The COVID-19 cohort included 211 critically ill patients admitted to ICU, 11 milder patients (not requiring corticosteroid treatment, ICU care, or high-flow oxygen at the time of sampling), and 16 after recovery (3–6 months after discharge). Samples from COVID-19 patients were collected between July 2020 and August 2021 in Uppsala and Gothenburg, Sweden. Control samples were collected from 17 healthy individuals, in Gdansk, Poland.

### Sample preparation

Sixteen milliters of blood was collected into BD Vacutainer® CPT™ Mononuclear Cell Preparation Tubes (BD). 1 ml of whole blood was saved for further DNA analysis. The tube was spun down within 4 h of collection; PBMC fraction was washed with PBS and cryopreserved in FBS (Fetal Bovine Serum) for long time storage at −150°C. Granulocytes together with red blood cells were recovered from the fraction below the gel of the CPT tube. The red blood cells were then lysed twice with 40 ml of 1× RBC lysis buffer (PharmLyse, BD) for 15 min at room temperature. The granulocytes were washed with PBS and cryopreserved in FBS (fetal bovine serum) for long time storage in −150°C. Cell number and viability was determined with Trypan blue and Countess II FL automated cell counter (Thermo Fisher). Approximately 200,000 cells were saved for DNA extraction.

### Preparation of cells for FACS

For FACS analysis, the PBMCs were mixed with FITC-labeled CD66b clone G10F5 (BD), APC-labeled CD15 clone HI98 (BD), and PE-CF594-labeled CD14 clone MφP9 (BD) and incubated for 20 min at 4°C. After incubation, PBMCs were washed with PBS and cell pellets were resuspended in 1 ml of PBS containing 3 mM EDTA. Before sorting, cells were filtered through 20 μm CellTrics (Sysmex) strainer to remove aggregates. The target cell populations were isolated using FACS Aria III (Becton Dickinson). Data were acquired and analyzed using BD FACSDiva™ Software (Becton Dickinson). Live cells were sorted based on their FSC and SSC, a singlets gate based on FSC-H and FSC-A was used to remove doublet cells. Neutrophils were identified as CD15+ and CD66b+; monocytes were defined based on their size and as CD14 +. Cells were sorted to achieve a purity of at least 96%. After sorting, cells were centrifuged and cell pellets were stored in −80°C freezer.

### DNA extraction

For samples with >50,000 cells the DNA was extracted using an in-house protocol previously described [5]. Cells were pelleted by centrifugation at 4000 rpm for 10 min and lysed with buffer containing 10 mM EDTA, 10 mM Tris–HCL (pH 7.9), 50 mM NaCl, and 1% N-Lauroylsarcosine sodium salt (Sigma) with 10 mg/ml proteinase K (Sigma). The DNA was then precipitated and resuspended in Low-TE. Samples with cell number between 10,000 to 50,000 cells were handled with an optimized cell lysis protocol. Lysis buffer without detergent was added to the cell pellet, together with 10 mg/ml proteinase K (Sigma), and incubated for 2 h in 50 °C. The cell lysate was then incubated in 95°C for 10 min to inactivate proteinase K. DNA from whole blood and granulocyte samples were extracted using the QIAmp DNA Blood Midi kit (Qiagen).

### Analysis of the level of loss of chromosome Y (LOY) using ddPCR

The LOY status was determined using ddPCR as described previously [7]. The DNA samples were pre-digested with HindIII (Thermo Fischer). Subsequently, 50 ng of the digested DNA was used in the analysis. The digested DNA was mixed with PCR supermix for probes without dUTP (BioRad) together with primers and probes for the AMELX/AMELY TaqMan-assay, number C_990000001_10 (Thermo Fisher). Quantification of the relative number of chromosomes X and Y in the sample was obtained by targeting the 6 bp sequence difference present between the AMELX and AMELY genes. Droplets were generated using the automated droplet generator (Bio-Rad), PCR was conducted using the T100 thermal cycler (Bio-Rad) and a QX200 Droplet reader (Bio-Rad) was used for the fluorescent measurements of droplets. The data was analyzed using the QuantaSoft software (Bio-Rad).

### Statistical analysis

Data was analyzed using Bayesian regression models via full Bayesian framework by calling *Stan* 2.21 [32] from *R* 4.1 [33] using the *brms* 2.16 [34] interface. Predictors and outcomes were centered and scaled. To reduce the influence of outliers and to better conform to the measured data, *Student’s t response distribution family with identity-link function* was used to model percent LOY outcomes and robust correlations. *Hurdle negative binomial distribution family with log-link function* was used to analyze FACS events counts. Models had no intercepts with indexing approach to predictors [35]. According to Stan recommendations [36] weakly informative priors were used for group-level effects, residual SD and group-level SD. P-values were produced by frequentist summary in *emmeans* 1.6 package [37]. Multiple testing correction was performed for tests with more than a single contrast and MVT-adjusted P-values are reported, adjusted using the multivariate t distribution with the same covariance structure as the estimates as implemented in *emmeans*. Medians of the posterior distribution and 80% and 95% HDI were plotted. Significant contrasts between groups were denoted as * if *p*≤0.05 and 95% HDI does not contain 0, and as ** if *p*≤0.01 and 99% HDI does not contain 0. Additionally, FACS events count data and percent LOY data were analyzed by Wilcoxon matched pairs signed-rank test with the significance level set to *p*≤0.05.

## Results

### Myeloid lineage cells from COVID-19 patients show the highest levels of LOY

**Fig. 1:**
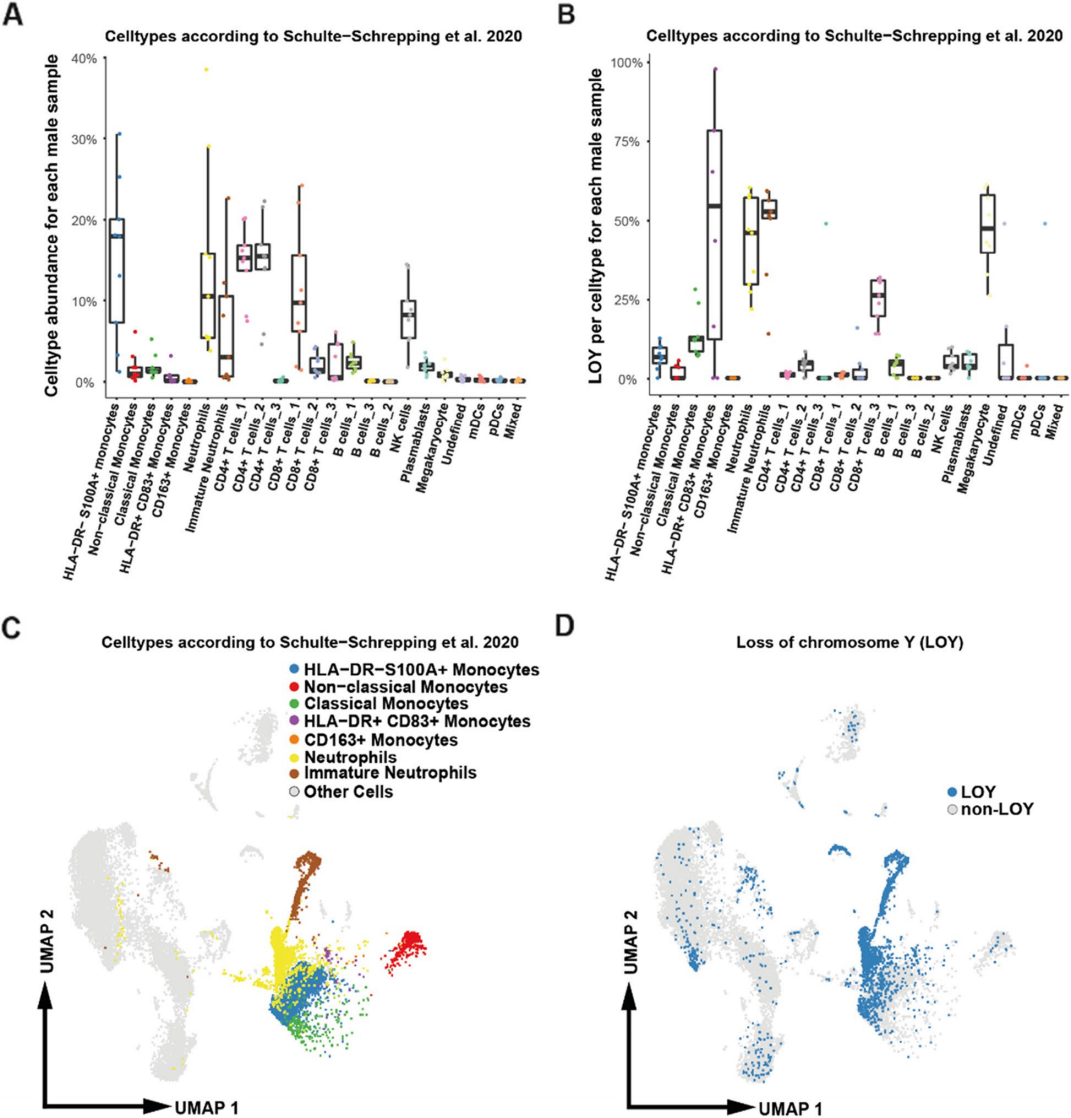
Distribution of cell types and cells with loss of chromosome Y (LOY) in PBMCs from critically ill patients with COVID-19. The dataset comes from Schulte-Schrepping et al. 2020 [19] and we used cell type annotation reported therein. Nine PBMCs samples from six male patients with WHO score >=5 were used for calculations. **A** Proportion of cell types in PBMCs; each data point represents a single sample. **B** Proportion of cells with LOY per cell type and per sample; each data point represents a single sample. **C** UMAP projection of scRNA-seq profiles, selected cell types are colored. **D** UMAP visualization of scRNA-seq profiles colored according to LOY status. Cells classified as LOY cells had no detectable expression from chromosome Y, as described [5]

Aiming to evaluate the frequency of cells with LOY and a possible impact of LOY on COVID-19 severity, we first re-analyzed the published single-cell RNA-seq dataset of leukocytes derived from COVID-19 patients, to which we added the LOY status. We applied the previously developed pipeline for LOY scoring in single-cells [5] in nine samples from six male patients with severe COVID-19 disease (WHO score 5–7), described in Schulte-Schrepping et al. 2020 [19]. In this re-analysis, we used two published cell-type annotations [19, 38]. Regardless of the applied method for cell annotation, we found concordant results suggesting that LOY was most frequent in neutrophils, immature neutrophils (also called low-density neutrophils, LDNs), monocytes, in particular monocyte-derived dendritic cells (HLA-DR+ CD83+ monocytes) and in megakaryocytes (Fig. 1 and Additional file 1: Fig. S1).

In order to extend the above initial analysis and verify the results, we collected blood samples from 211 critically ill male COVID-19 patients treated at ICU in two medical centers in Sweden (Uppsala and Gothenburg). The criterion for inclusion in our study as a critical COVID-19 patient was treatment at ICU (WHO score 6 or higher). The median age for this cohort was 64 years, ranging from 19 to 86 years. We also collected history of smoking habits, comorbidity data, as well as numerous clinical and biochemical parameters recorded during ICU treatment (Table 1). We further implemented a new protocol for Fluorescence-Activated Cell Sorting (FACS) enabling analysis of LOY in LDNs, granulocytes, monocytes, and PBMCs as well as in whole blood. The latter cell-sorting protocol was applied to 139 subjects out of 211 patients mentioned above. We also collected and sorted leukocytes from 11 mildly affected COVID-19 patients (WHO score 5 or lower) and 17 healthy controls. The overall design of our study is presented graphically in Additional file 1: Fig. S2. We used Bayesian statistical approach because of its robustness, e.g. its ability to reduce the influence of outliers and to use models better conforming to the measured data.

**Table 1:** Clinical characteristics of the control cases, mild and critically ill COVID-19 patients

**Controls**	***n*=17**
**Age,** median years (range)	71 (63–77)
**Smoking**	
Never	7 (41.2%)
Previous	8 (47%)
Ongoing	2 (11.8%)
**Mild patients**	***n*=11**
**Age,** median years (range)	74 (52–89)
**COVID-19 patients treated at intensive care unit (ICU)**	***n*=211**
**Age,** median years (range)	64 (19–86)
**Smoking**	
Never	119 (56.4%)
Previous	81 (38.4%)
Ongoing	11 (5.2%)
**Clinical characteristics of COVID-19 cohort treated at ICU**	
**History of comorbidities prior to ICU admission**	
BMI, *n*=199, median (IQR)	28.8 (25.8, 33)
Diabetes mellitus, *n*=191	57 (30%)
Vessel disease^a^, *n*=211	43 (20%)
Hypertension, *n*=191	110 (58%)
Pulmonary disease^b^, *n*=190	37 (19%)
**Measurements at admission to ICU**	
SOFA, *n*= 167, median (IQR)	8 (6, 10)
SAPS, *n*=161, median (IQR)	53 (47, 58)
CRP, *n*=186, median (IQR)	151 (85, 206)
Erythrocyte count, *n*=185, median (IQR)	4.39 (4, 4.73)
Thrombocyte count, *n*=184, median (IQR)	214 (168, 274)
Hemoglobin, *n*=190, median (IQR)	133 (122, 143)
Eotaxin level, *n*=173, median (IQR)	33 (25, 46)
P/F ratio, *n*=167, median (IQR)	18 (15.7, 20.9)
pH level, *n*=190, median (IQR)	7.46 (7.43, 7.48)
Oxygen saturation, *n*=190, median (IQR)	94.12 (92.75, 95.11)
IL-4 level, *n*=173, median (IQR)	2.55 (1.83, 3.62)
IL-5 level, *n*=173, median (IQR)	1 (1, 14)
**Clinical outcomes**	
Days at ICU, *n*=211, median (IQR)	10 (5, 18)
IMV at ICU, *n*=189	105 (56%)
Dialysis during ICU treatment, *n*=186	23 (12%)
Thromboembolic event during ICU stay^c^, *n*=187	27 (14%)
Critical illness^d^, *n*=183	32 (17%)
**WHO score,** *n*=190	
6 - Hospitalized, no mechanical ventilation or high-flow O_2_	77 (41%)
7 - Intubation and mechanical ventilation, P/F ≥ 150 or SpO_2_/FiO_2_ ≥ 200	-
8 - IMV, P/F <150 or vasopressors	11 (5.8%)
9 - IMV, P/F <150 and vasopressors, dialysis, or ECMO	69 (36%)
10 - Dead	33 (17%)

*IQR*, interquartile range; *SOFA*, Sequential Organ Failure Assessment score; *SAPS*, Simplified Acute Physiology Score; *CRP*, C-reactive protein; *IMV*, invasive mechanical ventilation

^a^Vessel disease was defined as the presence in the medical history of patients of any of the following conditions: coronary heart disease, stroke, diagnoses related to central vessels (aorta aneurysm, aorta stenosis), and diseases of peripheral vessels (intermittent claudication, skin ulcers of legs)

^b^Asthma, COPD, lung emphysema, pulmonary carcinoma, pulmonary fibrosis, cystic fibrosis, sarcoma, chronic interstitial pulmonary disease

^c^Pulmonary embolism, deep vein thrombosis, port thrombosis, cerebral infarction, mesenteric venous thrombosis, arterial thrombosis

^d^Diagnoses relating to the weakness after ICU treatment, neuropathy, or myopathy

**Fig. 2:**
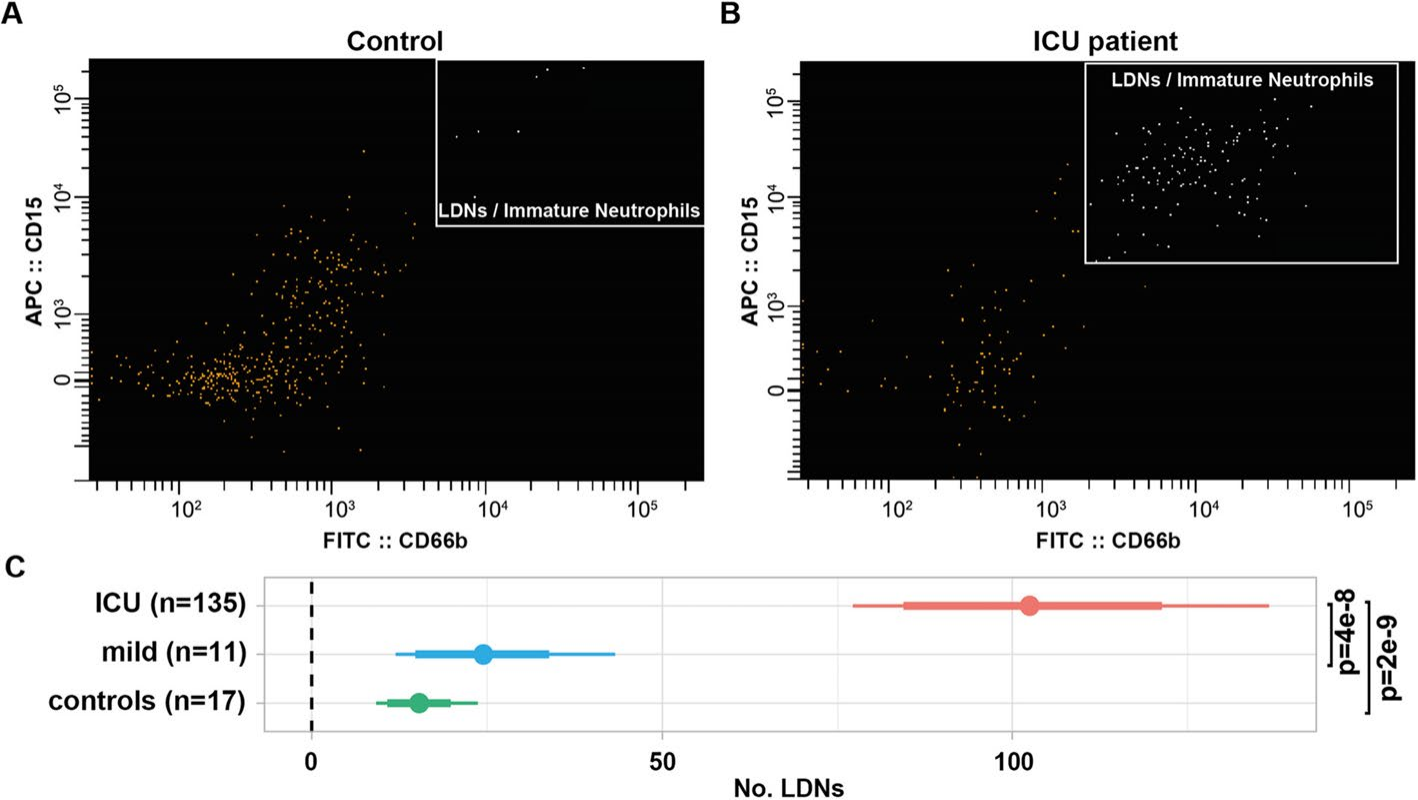
Differences in LDN counts between controls and critically ill patients (treated at ICU). FACS images showing the gating strategy employed to identify low-density neutrophils (LDNs, also called immature neutrophils), for representative control (**A**) and ICU patient (**B**) counted per 10,000 FACS events. PBMCs were gated for size and granularity, LDNs were identified as CD15+ (APC) and CD66b+ (FITC). **C** Age, age^2^, and smoking adjusted comparison of the numbers of LDNs in PBMCs between critically ill patients (ICU, *n*=135), milder patients (*n*=11), and healthy controls (*n*=17). Points show medians; thick and thin horizontal bars show 80% and 95% HDI. *P*-values are shown after MVT adjustment

The number of LDNs was the most variable among all analyzed cell types. Using FACS, we counted LDNs in critically ill COVID-19 patients, mild subjects, and healthy blood donors (Fig. 2A and B). The median number of LDNs (per 10,000 FACS events) in critically ill patients was 60, ranging from 1 to 458. The corresponding range of numbers for LDNs in milder COVID-19 patients (*n*=11) and healthy controls (*n*=17) were 1–70 and 1–73 per 10,000 FACS events, respectively. Figure 2C shows medians, 80% and 95% highest density intervals (HDI) for LDN cell counts, adjusted for age, age^2^, and smoking. The LDN cell counts in critically ill patients were significantly higher compared to healthy controls (median difference 87.0, 95% HDI 62.2 to 120.1, MVT-adjusted *p*=2e−9) and to milder COVID-19 patients (median difference 77.6, 95% HDI 50.4 to 109.7, MVT-adjusted *p*=4e−8).

**Fig. 3:**
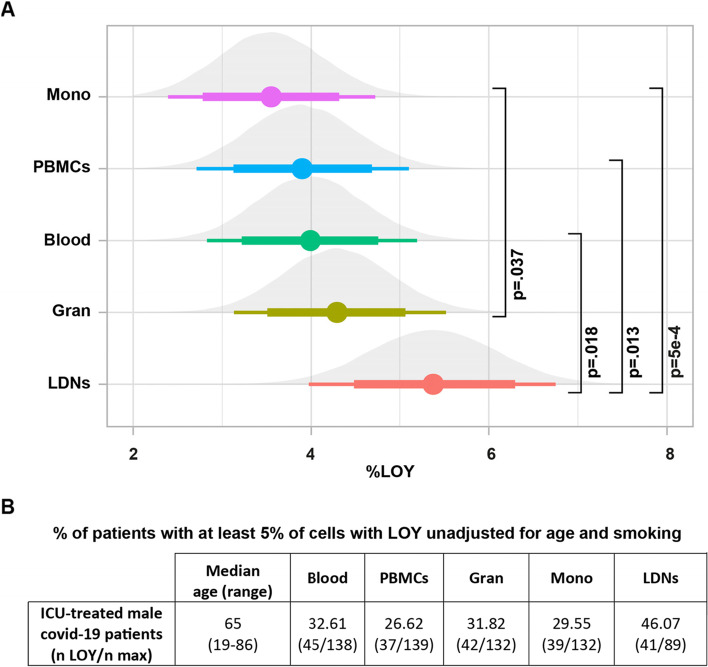
Comparison of %LOY across five cell populations for critically ill patients. **A** Age, age^2^, and smoking adjusted comparison of %LOY in monocytes, PBMCs, whole blood, granulocytes, and low-density neutrophils (LDNs) within ICU patients. Points show medians; thick and thin horizontal bars show 80% and 95% HDI. P-values are shown after MVT adjustment. **B** Patients with LOY in >5% of cells

The analysis of LOY status for all samples in this study was performed using droplet-digital PCR (ddPCR) as described [7]. The advantage of using ddPCR was a low input of DNA (~50 ng) required to perform the LOY measurement, which was particularly important for the analysis of LDNs from COVID-19 patients having low counts of these cells. Another advantage was a higher sensitivity in the lower range of measurements allowing to score LOY with a 5% cut-off (Fig. 3B). We were able to successfully detect LOY in DNA from LDNs sorted on FACS starting at about 10,000 cells. It should be stressed, however, that the number of successful measurements of LOY in LDNs was considerably lower than for other sources of DNA, due to the low number of LDNs in some COVID-19 patients as well as in controls and milder patients. Percentages of cells with LOY (%LOY) for all ICU patients in five different cell populations (LDNs, granulocytes, monocytes, PBMCs, and whole blood) were compared. Adjusting for age, age^2^, and smoking, %LOY in LDNs was significantly higher than in monocytes (median difference 1.83%, 95% HDI 0.94% to 2.72%, MVT-adjusted *p*=5e−4), in PBMCs (median difference 1.48%, 95% HDI 0.58% to 2.41%, MVT-adjusted *p*=0.013) and in blood (median difference 1.38%, 95% HDI 0.5% to 2.27%, MVT-adjusted *p*=0.018); and %LOY in granulocytes was significantly higher than in monocytes (median difference 0.75%, 95% HDI 0.22% to 1.26%, MVT-adjusted *p*=0.037) (Fig. 3A). Figure 3B and Additional file 1: Fig. S3 show unadjusted values, also pointing to the highest level of %LOY in LDNs compared with other cell types among critically ill patients. Over 46% of critically ill patients showed unadjusted %LOY above 5% cut-off in LDNs. The corresponding number for granulocytes was 32% of patients. These results, in particular LDN-derived, are linking LOY to severe COVID-19 phenotype.

### LOY associates to severity, mortality during ICU stay, and comorbidity in COVID-19 patients

**Fig. 4:**
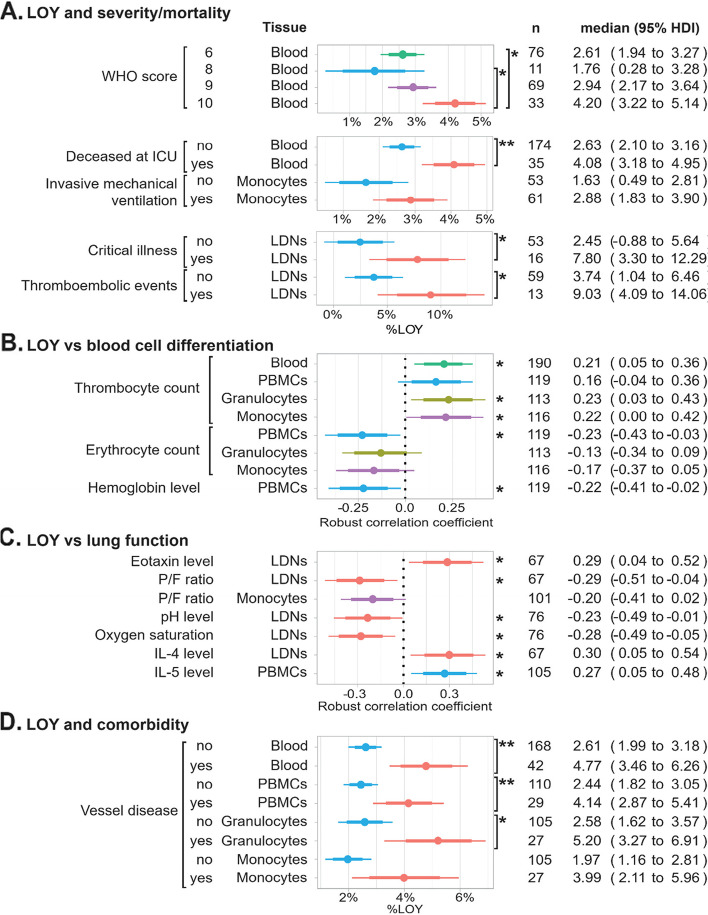
Associations between %LOY in blood or sorted populations of leukocytes and clinical variables, adjusting for age, age^2^, and smoking. Points show medians; thick and thin horizontal bars show 80%- and 95% HDI. *P*-values are MVT-adjusted and shown in the Results section. Significant tests were denoted by asterisks as * if *p*≤0.05 and 95% HDI does not contain 0, and as ** if *p*≤0.01 and 99% HDI does not contain 0

Next, we analyzed the relationship between %LOY in the previously mentioned cell fractions and blood DNA with the clinical parameters of patients in our cohort, adjusting for age, age^2^ and smoking. %LOY in blood of critically ill patients with WHO score 10 (i.e., deceased in COVID-19) was significantly higher than in patients with WHO score 6 (median difference 1.59%, 95% HDI 0.46% to 2.71%, MVT-adjusted *p*=0.025) and in patients with WHO score 8 (median difference 2.44%, 95% HDI 0.58% to 4.19%, MVT-adjusted *p*=0.035) (Fig. 4A). Overall, COVID-19 patients who deceased during treatment at ICU had significantly higher %LOY in blood than survivors (median difference 1.46%, 95% HDI 0.47% to 2.43%, *p*=0.0036). %LOY in LDNs was significantly higher in patients who suffered from the critical illness assessed by manifestation of post-ICU symptoms such as neuropathy or myopathy (median difference 5.33%, 95% HDI 0.56% to 10.3%, *p*=0.031) as well as in patients who suffered from thromboembolic events during ICU treatment (median difference 5.30%, 95% HDI 0.46% to 10.2%, *p*=0.033) compared to patients without such complications. Moreover, we observed a trend that %LOY was higher in monocytes of patients requiring invasive mechanical ventilation (median difference 1.25%, 95% HDI −0.01% to 2.48%, *p*=0.05) (Fig. 4A). Taken together, these results suggest that LOY in whole blood as well as in selected myeloid cells (monocytes and LDNs) is significantly associated with COVID-19 severity classified by the WHO score and mortality from COVID-19.

Previous epidemiological studies showed an association of LOY with differentiation of blood cells; that is thrombocyte- and erythrocyte cell counts were positively and negatively associated with LOY, respectively [39, 40]. In particular, binding sites of FLI1, a fate-determining factor promoting hematopoietic stem cell differentiation into thrombocytes rather than erythrocytes, showed a significant heritability enrichment of LOY GWAS signals using ChIP-seq data [39]. This effect of LOY might be important for the clinical outcome of critically ill male COVID-19 patients that display LOY, since increased counts of thrombocytes, hyper-coagulation, and thromboembolic events are contributing to the high mortality in COVID-19 [22, 23]. We have therefore analyzed our critically ill patients for the correlation of %LOY in five cell populations with the cell counts of thrombocytes and erythrocytes as well as hemoglobin levels, adjusting for age, age^2^, and smoking (Fig. 4B and Table 1). The cell counts for thrombocytes and erythrocytes as well as hemoglobin measurements were collected on the day of admission to ICU and followed during the entire length of ICU stay. Thrombocyte count was significantly positively correlated with %LOY in blood (robust correlation coefficient *R*=0.21, 95% HDI 0.05 to 0.36) and in granulocytes (*R*=0.23, 95% HDI 0.03 to 0.43). %LOY in PBMCs showed a significant negative correlation with erythrocyte counts (*R*= −0.23, 95% HDI −0.43 to −0.03). These results agree with the above-described differences in %LOY between patients with and without thromboembolic events and are concordant with literature showing the effect of LOY on blood cell differentiation [39, 40]. Finally, %LOY in PBMCs was also significantly negatively correlated with hemoglobin level (*R*= −0.22, 95% HDI −0.41 to −0.02).

Other parameters monitored during the ICU stay were factors reflecting the condition of the lungs. Interestingly, our analysis revealed that %LOY is correlated with a range of these parameters, corroborating the hypothesis that LOY is linked to the COVID-19 severity. We have found a significant positive correlation between %LOY in LDNs and Eotaxin-1 (CCL11) levels (*R*=0.29, 95% HDI 0.04 to 0.52) as well as IL-4 levels (*R*=0.30, 95% HDI 0.05 to 0.54). We also observed a significant positive correlation between %LOY in PBMCs and levels of IL-5 (*R*=0.27, 95% HDI 0.05 to 0.48). Moreover, a significant negative correlation was observed between %LOY and respiratory performance of lungs measured by P/F ratio (*R*= −0.29, 95% HDI -0.51 to −0.04 for LDNs; *R*= −0.20, 95% HDI −0.41 to 0.02 for monocytes), pH level (*R*= −0.23, 95% HDI −0.45 to −0.01 for LDNs) and oxygen saturation (*R*= −0.28, 95% HDI −0.49 to −0.05 for LDNs) (Fig. 4C).

We also tested for differences in %LOY related to comorbidities prior to ICU admission. The comorbidity group Vessel Disease (VD), was defined as the presence in the history of patients any of the following conditions: coronary heart disease, stroke, diagnoses related to central vessels (aorta aneurysm, aorta stenosis), and diseases of peripheral vessels (intermittent claudication, skin ulcers of legs). Figure 4D shows that, compared to patients without VD, adjusting for age, age^2^, and smoking, %LOY was significantly higher for patients with a history of VD in blood (median difference 2.16%, 95% HDI 0.74% to 3.7%, *p*=0.004), in PBMCs (median difference 1.70%, 95% HDI 0.43% to 2.98%, *p*=0.0089), in granulocytes (median difference 2.62%, 95% HDI 0.53% to 4.55%, *p*=0.012) and a trend in monocytes (median difference 2.02%, 95% HDI 0.05% to 4.12%, *p*=0.05). Thus, we show that %LOY in the blood is associated with common comorbidities present among COVID-19 patients treated at ICUs (Fig. 4D).

### The level of LOY is dynamic and decreases in COVID-19 patients at the recovery stage

**Fig. 5:**
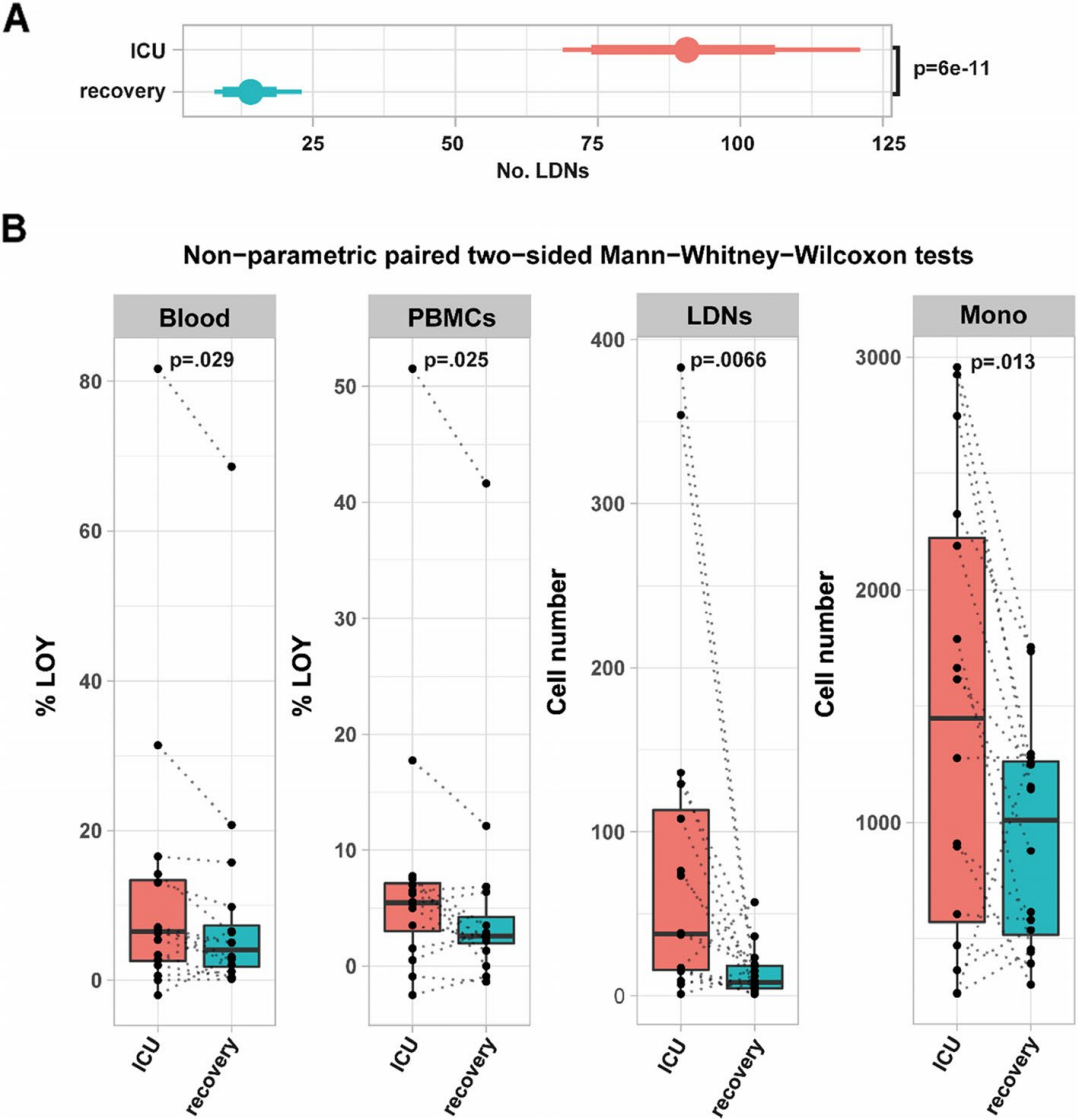
Leukocyte counts and LOY status for patients during ICU treatment and during recovery. **A** Age, age^2^, and smoking adjusted comparison of the numbers of low-density neutrophils (LDNs) at the intensive care unit (ICU, *n*=135) and 3–6 months after the discharge from ICU (recovery, *n*=16). Points denote medians; thick and thin horizontal bars show 80% and 95% HDI, respectively. **B** Paired comparison of unadjusted loss of chromosome Y percentages (%LOY) in whole blood and PBMC, and numbers of LDNs and monocytes cells in PBMCs in 16 patients, where both ICU and recovery samples were available. Each dotted line connects the ICU and recovery data within the same patient. Boxplots show median, IQR as hinges and largest values no further than 1.5*IQR away as whiskers. Unadjusted *p*-values from paired two-sided Mann-Whitney-Wilcoxon non-parametric tests are shown

We also studied follow-up blood samples from 16 critically ill patients who recovered from the disease. The median number of days when the follow-up samples were collected (from the date of first sampling), was 119 days, ranging from 93 to 143. These samples were processed in the same way as the described above for 139 critically ill patients, with cell sorting and separate analysis of LOY for each fraction. Figure 5A shows medians, 80% and 95% HDI for estimates of LDN cell counts per 10,000 FACS events, adjusted for age, age^2^, and smoking, demonstrating a radical decrease in the follow-up samples (median difference 76.4, 95% HDI 55.5 to 104, *p*=6e−11). Paired comparisons of unadjusted cell numbers from LDNs and monocytes in 16 patients, where both ICU and recovery samples were available, also showed a statistically significant decrease in the follow-up samples for LDNs (*p*=0.0066) and monocytes (*p*=0.013) using non-parametric paired Mann-Whitney-Wilcoxon test (Fig. 5B).

To investigate the LOY level in convalescent patients, we further performed paired LOY analysis in the five cell populations in 16 patients, where both ICU and recovery samples were available. Because the number of sorted LDNs from recovery samples was too low for DNA isolation (below 10,000 cells isolated with FACS from 16 ml of blood), we studied LOY only in the four other cell types; i.e., blood, PBMCs, granulocytes, and monocytes. Paired comparisons of unadjusted %LOY in blood (*p*=0.029) and PBMCs (*p*=0.025) showed a significant decrease in samples taken at the recovery stage (Fig. 5B). In conclusion, the results from comparisons between ICU and recovery samples suggest the overall decrease of cell numbers that are primarily associated with the critical course of COVID-19 (LDNs and monocytes) as well as the overall decrease in the load of cells with LOY. The latter result underscores the dynamic character of aberrant cell clones with LOY that are present in COVID-19 patients.

## Discussion

The COVID-19 pandemic is the most serious new global disease of this century with a strong male bias. Understanding the male bias in the severity and mortality of COVID-19 is a central, albeit still poorly explored aspect of COVID-19 pathogenesis, which might be important for future strategies aiming at better prevention and treatment of critically ill patients [29]. We suggest here that LOY might be a new factor underlying the higher severity and mortality of this infection in males, which is in line with another preliminary report [41]. Our results link LOY to an acute, often life-threatening viral infection, representing a step forward in the understanding of the role of LOY in the susceptibility to disease, especially in relation to the dysfunction of the immune system.

The available evidence for a higher morbidity and mortality of men in COVID-19 aligns with pre-existing chronic diseases that are either more common and/or occur earlier in life among males. However, this is unlikely to be the whole explanation behind male bias for COVID-19 severity. Our results regarding the clonal expansions of LDNs and coexisting high %LOY in these cells among males with the critical disease might also be a part of the explanation. We focused this study on the correlations between LOY and phenotype of critically ill patients with COVID-19 treated at ICUs. Such patients are very often burdened with multiple comorbidities, many of which have previously been associated with LOY. Thus, it is not straightforward to disentangle possible direct effects of LOY on the immune system from the effects of pre-existing diseases. In this context, the most convincing future investigation of the relationship between LOY and morbidity/mortality from COVID-19 should be to restrict the analysis to men treated at ICUs who are free of these pre-existing conditions.

We present several lines of evidence linking LOY to severe COVID-19. The first is the highest levels of LOY in cells of myeloid origin known to be crucial for the development of severe disease, especially in LDNs and granulocytes, the former being aberrant, pathological cells present in high numbers in peripheral blood from critically ill COVID-19 patients. Secondly, we correlate LOY to multiple clinical variables, most importantly severity of the disease according to WHO score and mortality during ICU treatment. Moreover, we report increased counts of thrombocytes and decrease counts of erythrocytes, which is in line with previous reports of LOY effects, shown in non-COVID-19 population-based cohorts [39, 40]. Thromboembolic complications also correlated with increased levels of LOY in LDNs and monocytes, which may contribute to the severity of COVID-19 disease in males with LOY, eventually leading to organ failure, in particular affecting the lungs. Thirdly, cell clones with LOY are transient and dynamic when samples that were taken from the same patients during ICU stay and 3–6 months later are compared. As far as we know, this is the first example of the dynamic character of cell clones with LOY that is associated with an acute disease.

We have previously reported [5] and show it here again in the context of COVID-19 that LOY can be scored on single-cell RNA-seq data, allowing analysis of the impact of this large mutation on transcription in selected leukocyte populations. Using this approach, we re-analyzed published scRNA-seq data [19] showing that LDNs (or immature neutrophils), which play a key role in the pathogenesis of COVID-19, exhibit high levels of LOY in critically ill male COVID-19 patients (Fig. 1). It should be mentioned here that scRNA-seq has frequently been used in studies of COVID-19 and there exist numerous additional publicly available scRNA-seq datasets, which will facilitate follow-up and extension of our results in additional cohorts. Our results from scRNA-seq were confirmed at the DNA level, as we demonstrated that LDNs from critically ill patients displayed LOY, representing 46% of the studied COVID-19 patients (Fig. 3). Comparing the literature and considering the young age of patients in our cohort (median age 64 years), this is a high number of subjects showing LOY in blood DNA. This observation is also in line with other results suggesting that the myeloid lineage is the most affected by LOY [5, 9].

As mentioned above, epidemiological studies have associated LOY with myeloid cell differentiation [39, 40]. A high neutrophil-to-lymphocyte ratio is also a recognized marker of COVID-19 severity [42]. Considering the high percentage of LOY cells among neutrophils of COVID-19 patients, it is possible that the clonal expansion is at least partially driven by LOY and functional disturbance of these cells might also be a consequence of LOY. The decreasing level of LOY in blood and PBMCs from recovering patients might be a useful biomarker of severe disease and/or successful recovery. Neutrophils are the cell type with the second highest daily turnover (after erythrocytes) and are intensively produced during infections [43]. Therefore, the progenitors for neutrophils are highly prone to age-related accumulation of various post-zygotic mutations, among them LOY and mutations causing clonal hematopoiesis of indeterminate potential (CHIP). In line with the above, we have recently shown that LOY in monocytes is accompanied by pathogenic somatic mutations in genes associated with DNA methylation (*TET2*, *DNMT3A*), transcription regulation (*ASXL1*), and DNA repair (*TP53*) [9]. Based on large-scale population studies, 10% of people older than 65 are affected by CHIP mutations [44]. Assuming that CHIP mutations and LOY co-exist in some clonally expanded LDNs or granulocytes/monocytes in critically ill COVID-19 patients, different CHIP variants might provide diverse proliferative advantage to such clones and this subject should be studied further. Moreover, a trend suggesting a higher total count of mosaic chromosomal aberrations in blood that is associated with hospitalization for COVID-19 has been reported [45]. The male gender is a risk factor in critical COVID-19 and a link between genetic variants of the Y chromosome and severe COVID-19 has been reported [46, 47]. However, a recent larger investigation, in which haplogroups on the Y chromosome were defined based on the analysis of SNP arrays, showed no significant association between COVID-19 and the Y-haplogroups [48]. Thus, the impact of Y-haplogroups on male immunity and its role in COVID-19 severity remains an open question and further studies are needed. Yet another hypothesis that could contribute to explaining the greater mortality in men with COVID-19 relates to androgen levels. It was reported that prostate cancer patients treated with androgen-deprivation therapy were less susceptible to SARS-CoV-2 infection [49], suggesting that low testosterone levels could be protective against COVID-19. However, a recent review highlights inconsistency in research showing the effects of testosterone in COVID-19 [50], and the question of how androgens influence COVID-19 severity in males requires further analyses.

Male predominance for critical COVID-19 is consistent with a similar bias for prior severe acute respiratory syndrome (SARS) and Middle East respiratory syndrome (MERS) epidemics (caused by SARS-CoV and MERS-CoV viruses, respectively) [51–53]. Moreover, male-female differences have also been reported for seasonal influenza A virus infections in Australia and Japan [54, 55]. Consequently, the importance of our results is not limited to COVID-19, and further research should be conducted in the context of this disease as well as other common viral infections.

## Conclusions

We performed the analysis of the immune system cells affected by LOY, concentrating on men with critical COVID-19. Our results revealed that the highest levels of LOY were found in the cells of the myeloid lineage, particularly in LDNs, which are crucial for the development of severe COVID-19 phenotype. Importantly, we showed that LOY was associated with the severity of the disease according to increasing WHO score and mortality during ICU treatment. We also present the first example of the dynamic nature of cell clones with LOY that are associated with acute infectious disease. Moreover, our results suggest that LOY could be considered as predictive biomarker in identifying patients at high risk of developing a critical course of COVID-19. Overall, the data support the link between LOY and emergency myelopoiesis as well as the role of LOY in modulating the severity of COVID-19 disease. We further provide new insight into the response of the male immune system to COVID-19, which might also be relevant for other common viral infections showing a similar male bias.

## Supplementary Information

**Additional file 1: Fig. S1.** The proportion of selected populations of PBMCs in critical COVID-19 patients. **Fig. S2.** Schematic presentation of the overall study design. **Fig. S3.** Comparison of %LOY across five cell populations for patients during ICU treatment.

## Data Availability

Clinical data from Uppsala University Hospital (PronMed) cohort is available through the SciLifeLab data repository after securing ethical permission and appropriate data access agreements (10.17044/scilifelab.14229410). The data from Sahlgrenska University Hospital cohort from Gothenburg is available from the Dr. Magnus Gisslen (magnus.gisslen@infect.gu.se) upon reasonable request.

## References

[1] Forsberg LA, Rasi C, Malmqvist N, Davies H, Pasupulati S, Pakalapati G (2014). Mosaic loss of chromosome Y in peripheral blood is associated with shorter survival and higher risk of cancer. Nature Genetics..

[2] Dumanski JP, Lambert JC, Rasi C, Giedraitis V, Davies H, Grenier-Boley B (2016). Mosaic Loss of Chromosome Y in Blood Is Associated with Alzheimer Disease. Am J Hum Genet..

[3] Thompson D, Genovese G, Halvardson J, Ulirsch J, Wright D, Terao C (2019). Genetic predisposition to mosaic Y chromosome loss in blood. Nature..

[4] Forsberg L, Halvardson J, Rychlicka E, Danielsson M, Torabi Moghadam B, Mattisson J (2019). Mosaic loss of chromosome Y (LOY) in leukocytes matters. Nature Genetics..

[5] Dumanski J, Halvardson J, Davies H, Rychlicka-Buniowska E, Mattisson J, Torabi Moghadam B (2021). Immune cells lacking Y chromosome show dysregulation of autosomal gene expression. Cell Mol Life Sci..

[6] Haitjema S, Kofink D, van Setten J, van der Laan S, Schoneveld A, Eales J (2017). Loss of Y Chromosome in Blood Is Associated with Major Cardiovascular Events during Follow-up in Men after Carotid Endarterectomy. Circulation: Cardiovascular. Genetics..

[7] Danielsson M, Halvardson J, Davies H, Torabi Moghadam B, Mattisson J, Rychlicka-Buniowska E (2020). Longitudinal changes in the frequency of mosaic chromosome Y loss in peripheral blood cells of aging men varies profoundly between individuals. Eur J Hum Genet..

[8] Dumanski JP, Rasi C, Lonn M, Davies H, Ingelsson M, Giedraitis V (2015). Smoking is associated with mosaic loss of chromosome Y. Science..

[9] Ljungstrom V, Mattisson J, Halvardson J, Pandzic T, Davies H, Rychlicka-Buniowska E (2022). Loss of Y and clonal hematopoiesis in blood-two sides of the same coin?. Leukemia..

[10] Ganster C, Kampfe D, Jung K, Braulke F, Shirneshan K, Machherndl-Spandl S (2015). New data shed light on Y-loss-related pathogenesis in myelodysplastic syndromes. Genes Chromosomes Cancer..

[11] Loftfield E, Zhou W, Graubard BI, Yeager M, Chanock SJ, Freedman ND (2018). Predictors of mosaic chromosome Y loss and associations with mortality in the UK Biobank. Sci Rep..

[12] Loftfield E, Zhou W, Yeager M, Chanock SJ, Freedman ND, Machiela MJ (2019). Mosaic Y Loss Is Moderately Associated with Solid Tumor Risk. Cancer Res..

[13] Persani L, Bonomi M, Lleo A, Pasini S, Civardi F, Bianchi I (2012). Increased loss of the Y chromosome in peripheral blood cells in male patients with autoimmune thyroiditis. Journal of autoimmunity..

[14] Lleo A, Oertelt-Prigione S, Bianchi I, Caliari L, Finelli P, Miozzo M (2013). Y chromosome loss in male patients with primary biliary cirrhosis. Journal of autoimmunity..

[15] Grassmann F, Kiel C, den Hollander A, Weeks D, Lotery A, Cipriani V (2019). Y chromosome mosaicism is associated with age-related macular degeneration. European Journal of Human Genetics..

[16] Mattisson J, Danielsson M, Hammond M, Davies H, Gallant CJ, Nordlund J (2021). Leukocytes with chromosome Y loss have reduced abundance of the cell surface immunoprotein CD99. Scientific Reports..

[17] Zhu N, Zhang D, Wang W, Li X, Yang B, Song J (2020). A Novel Coronavirus from Patients with Pneumonia in China, 2019. N Engl J Med..

[18] Hu B, Guo H, Zhou P, Shi ZL (2021). Characteristics of SARS-CoV-2 and COVID-19. Nat Rev Microbiol..

[19] Schulte-Schrepping J, Reusch N, Paclik D, Bassler K, Schlickeiser S, Zhang B (2020). Severe COVID-19 Is Marked by a Dysregulated Myeloid Cell Compartment. Cell..

[20] Ren X, Wen W, Fan X, Hou W, Su B, Cai P (2021). COVID-19 immune features revealed by a large-scale single-cell transcriptome atlas. Cell..

[21] Huckriede J, Anderberg SB, Morales A, de Vries F, Hultström M, Bergqvist A (2021). Evolution of NETosis markers and DAMPs have prognostic value in critically ill COVID-19 patients. Sci Rep..

[22] Spiezia L, Boscolo A, Poletto F, Cerruti L, Tiberio I, Campello E (2020). COVID-19-Related Severe Hypercoagulability in Patients Admitted to Intensive Care Unit for Acute Respiratory Failure. Thromb Haemost..

[23] Kollias A, Kyriakoulis KG, Dimakakos E, Poulakou G, Stergiou GS, Syrigos K (2020). Thromboembolic risk and anticoagulant therapy in COVID-19 patients: emerging evidence and call for action. Br J Haematol..

[24] Busch MH, Timmermans S, Nagy M, Visser M, Huckriede J, Aendekerk JP (2020). Neutrophils and Contact Activation of Coagulation as Potential Drivers of COVID-19. Circulation..

[25] Zuo Y, Zuo M, Yalavarthi S, Gockman K, Madison JA, Shi H (2021). Neutrophil extracellular traps and thrombosis in COVID-19. J Thromb Thrombolysis..

[26] Swedish Intensive Care Database (SIR). 2021, September 9 [Available from: https://www.icuregswe.org/en/data%2D%2Dresults/covid-19-in-swedish-intensive-care/.

[27] Peckham H, de Gruijter NM, Raine C, Radziszewska A, Ciurtin C, Wedderburn LR (2020). Male sex identified by global COVID-19 meta-analysis as a risk factor for death and ITU admission. Nat Commun..

[28] Gebhard C, Regitz-Zagrosek V, Neuhauser HK, Morgan R, Klein SL (2020). Impact of sex and gender on COVID-19 outcomes in Europe. Biol Sex Differ..

[29] Scully EP, Haverfield J, Ursin RL, Tannenbaum C, Klein SL (2020). Considering how biological sex impacts immune responses and COVID-19 outcomes. Nat Rev Immunol..

[30] Asano T, Boisson B, Onodi F, Matuozzo D, Moncada-Velez M, Maglorius Renkilaraj MRL (2021). X-linked recessive TLR7 deficiency in ~1% of men under 60 years old with life-threatening COVID-19. Sci Immunol..

[31] Bastard P, Rosen LB, Zhang Q, Michailidis E, Hoffmann HH, Zhang Y (2020). Autoantibodies against type I IFNs in patients with life-threatening COVID-19. Science..

[32] Stan Development Team. RStan: the R interface to Stan. R package version 2.21.2. 2020 [Available from: http://mc-stan.org/.

[33] R Core Team. R: A language and environment for statistical computing. R Foundation for Statistical Computing. Vienna; Austria; 2021. [Available from: https://www.R-project.org/

[34] Bürkner P-C (2017). brms: An R Package for Bayesian Multilevel Models Using Stan. Journal of Statistical Software..

[35] McElreath R (2020). Statistical Rethinking. A Bayesian Course with Examples in R and STAN.

[36] Gelman A. Prior Choice Recommendations. In: Stan-dev/stan. Ed. GitHub 2019 [Available from: https://github.com/stan-dev/stan/wiki/Prior-Choice-Recommendations.

[37] Lenth RV. emmeans: Estimated Marginal Means, aka Least-Squares Means. R package version 1.6.3. 2021 [Available from: https://CRAN.R-project.org/package=emmeans.

[38] Monaco G, Lee B, Xu W, Mustafah S, Hwang YY, Carre C (2019). RNA-Seq Signatures Normalized by mRNA Abundance Allow Absolute Deconvolution of Human Immune Cell Types. Cell Rep..

[39] Terao C, Momozawa Y, Ishigaki K, Kawakami E, Akiyama M, Loh PR (2019). GWAS of mosaic loss of chromosome Y highlights genetic effects on blood cell differentiation. Nat Commun..

[40] Lin SH, Loftfield E, Sampson JN, Zhou W, Yeager M, Freedman ND (2020). Mosaic chromosome Y loss is associated with alterations in blood cell counts in UK Biobank men. Sci Rep..

[41] Pérez-Jurado LA, Cáceres A, Esko T, de Heredia ML, Quintela I, Cruz R (2022). Clonal chromosomal mosaicism and loss of chromosome Y in men are risk factors for SARS-CoV-2 vulnerability in the elderly. medRxiv..

[42] Carissimo G, Xu W, Kwok I, Abdad MY, Chan YH, Fong SW (2020). Whole blood immunophenotyping uncovers immature neutrophil-to-VD2 T-cell ratio as an early marker for severe COVID-19. Nat Commun..

[43] Sender R, Milo R (2021). The distribution of cellular turnover in the human body. Nature medicine..

[44] Jaiswal S, Fontanillas P, Flannick J, Manning A, Grauman PV, Mar BG (2014). Age-related clonal hematopoiesis associated with adverse outcomes. N Engl J Med..

[45] Zekavat SM, Lin SH, Bick AG, Liu A, Paruchuri K, Wang C (2021). Hematopoietic mosaic chromosomal alterations increase the risk for diverse types of infection. Nature medicine..

[46] Delanghe JR, De Buyzere ML, De Bruyne S, Van Criekinge W, Speeckaert MM (2020). The potential influence of human Y-chromosome haplogroup on COVID-19 prevalence and mortality. Ann Oncol..

[47] Ibrahim M, Salih A (2021). The Y chromosome ancestry marker R1b1b2: a surrogate of the SARS-CoV-2 population affinity. Hum Genome Var..

[48] Degenhardt F, Ellinghaus D, Juzenas S, Lerga-Jaso J, Wendorff M, Maya-Miles D (2022). Detailed stratified GWAS analysis for severe COVID-19 in four European populations. Hum Mol Genet..

[49] Montopoli M, Zumerle S, Vettor R, Rugge M, Zorzi M, Catapano CV (2020). Androgen-deprivation therapies for prostate cancer and risk of infection by SARS-CoV-2: a population-based study (N = 4532). Ann Oncol..

[50] Lo SP, Hsieh TC, Pastuszak AW, Hotaling JM, Patel DP (2022). Effects of SARS CoV-2, COVID-19, and its vaccines on male sexual health and reproduction: where do we stand?. Int J Impot Res..

[51] Karlberg J, Chong DS, Lai WY (2004). Do men have a higher case fatality rate of severe acute respiratory syndrome than women do?. Am J Epidemiol..

[52] Leong HN, Earnest A, Lim HH, Chin CF, Tan C, Puhaindran ME (2006). SARS in Singapore--predictors of disease severity. Ann Acad Med Singap..

[53] Alghamdi IG, Hussain II, Almalki SS, Alghamdi MS, Alghamdi MM, El-Sheemy MA (2014). The pattern of Middle East respiratory syndrome coronavirus in Saudi Arabia: a descriptive epidemiological analysis of data from the Saudi Ministry of Health. Int J Gen Med..

[54] Wong KC, Luscombe GM, Hawke C (2019). Influenza infections in Australia 2009-2015: is there a combined effect of age and sex on susceptibility to virus subtypes?. BMC Infect Dis..

[55] Eshima N, Tokumaru O, Hara S, Bacal K, Korematsu S, Tabata M (2011). Sex- and age-related differences in morbidity rates of 2009 pandemic influenza A H1N1 virus of swine origin in Japan. PLoS One..

