# Supplementary material for: Loss of Y in leukocytes as a risk factor for critical COVID-19 in men
Source: Genome Med. 2022 Dec 14;14:139. doi: 10.1186/s13073-022-01144-5 (PMC9747543; doi:10.1186/s13073-022-01144-5)
Supplement: Supplementary file 1 — Additional file 1: Fig. S1. The proportion of selected populations of PBMCs in critical COVID-19 patients. Fig. S2. Schematic presentation of the overall study design. Fig. S3. Comparison of %LOY across five cell populations for patients during ICU treatment. [file 13073_2022_1144_MOESM1_ESM.docx]

**Additional files**

**Loss of Y in leukocytes as a risk factor for critical COVID-19 in men**

Bożena Bruhn-Olszewska, Hanna Davies, Daniil Sarkisyan, Ulana Juhas, Edyta Rychlicka-Buniowska, Magdalena Wójcik^2^, Monika Horbacz, Marcin Jąkalski, Paweł Olszewski, Jakub O. Westholm, Agata Smialowska, Karol Wierzba, Åsa Torinsson Naluai, Niklas Jern, Lars-Magnus Andersson, Josef D. Järhult, Natalia Filipowicz, Eva Tiensuu Janson, Sten Rubertsson, Miklós Lipcsey, Magnus Gisslén, Michael Hultström, Robert Frithiof, Jan P. Dumanski

**Figure S1**

**Figure S2**

**Figure S3**


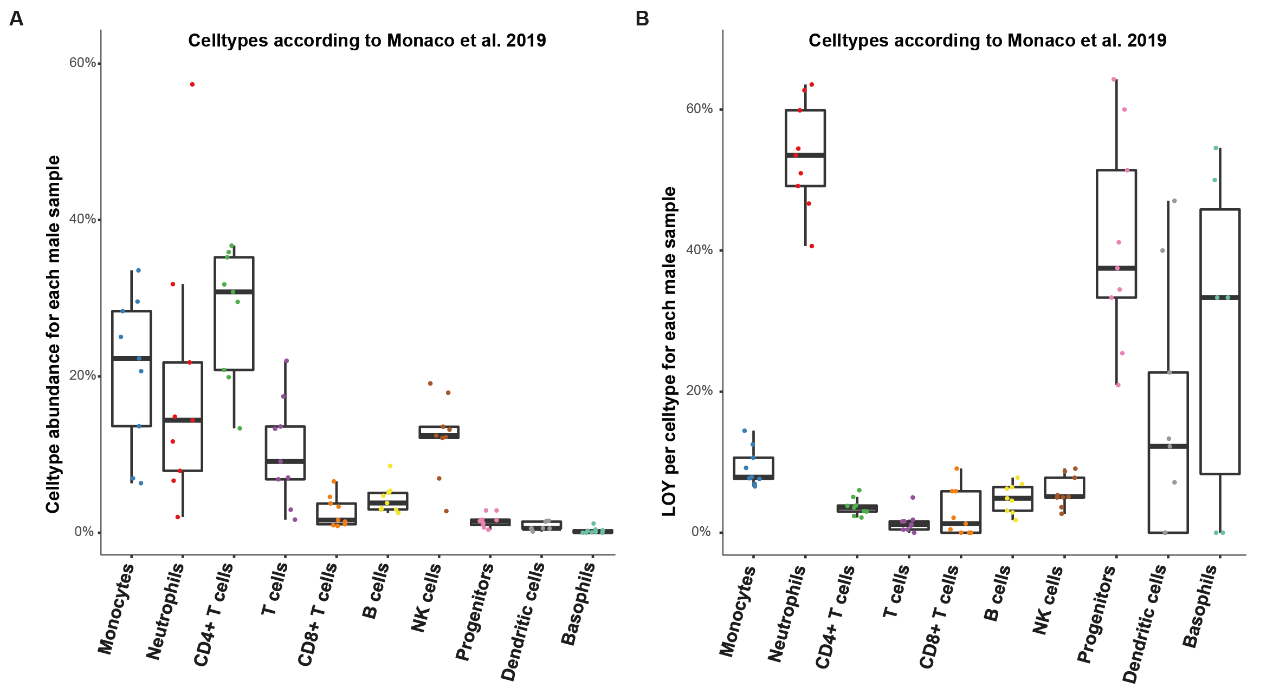


**Fig. S1. The proportion of selected populations of PBMCs in critical COVID-19 patients.** The dataset comes from Schulte-Schrepping et al. 2020. Cell types were annotated using SingleR according to Monaco et al. 2019. Nine PBMC samples from six male patients with WHO score >=5 were used for calculations. **A)** Proportion of cell types in PBMCs from COVID-19 patients; each data point represents a single sample. **B)** Proportion of cells with LOY per cell type and per sample; each data point represents a single sample. Cells classified as LOY-cells had no detectable expression from chromosome Y, as described (Dumanski, et al. 2021).


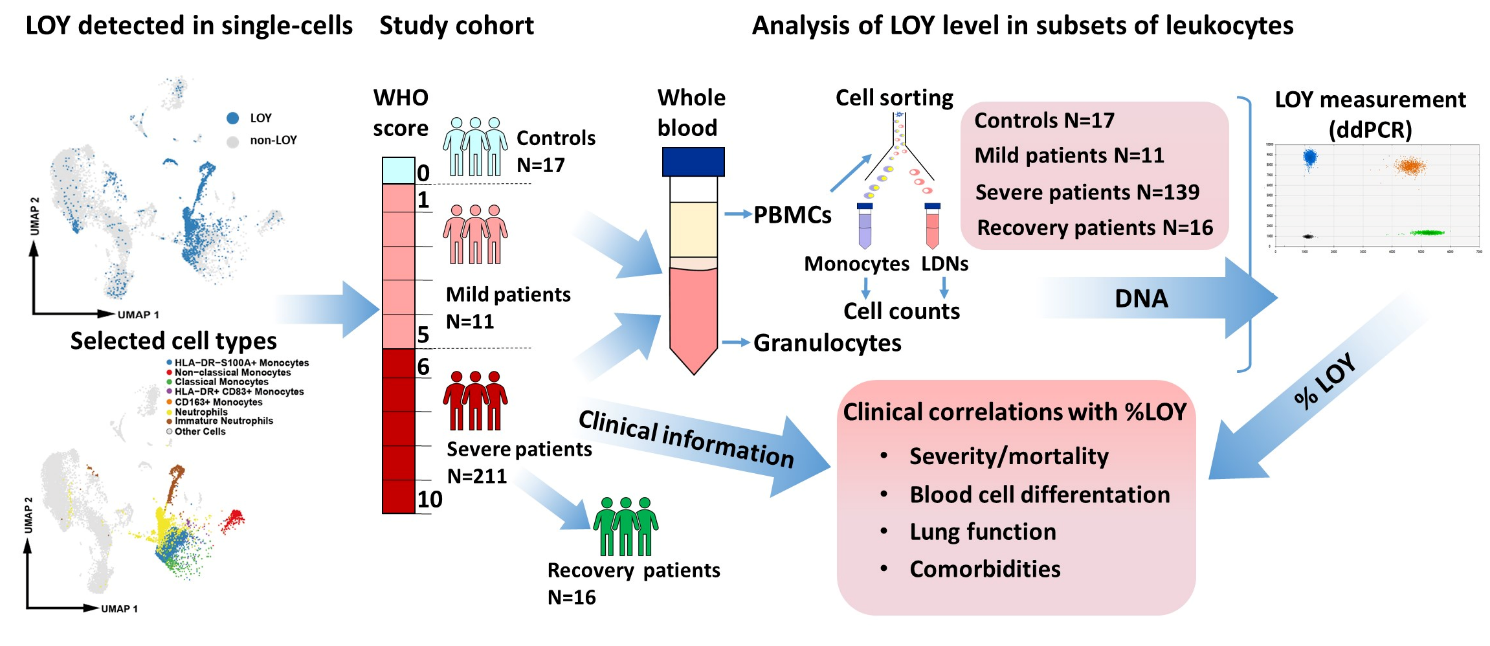


**Fig. S2. Schematic presentation of the overall study design**. The project started with a re-analysis of the published single-cell RNA dataset. These results were followed by the recruitment and analysis of 211 ICU patients (WHO score 6-10), 11 mild patients (WHO score 1-5), 17 healthy controls, and 16 recovery patients. PBMCs were isolated by density centrifugation and subjected to Fluorescence-Activated Cell Sorting (FACS). DNA was isolated from whole blood and selected subsets of leukocytes, and the measurement of LOY level was carried out using the digital droplet PCR (ddPCR) method.


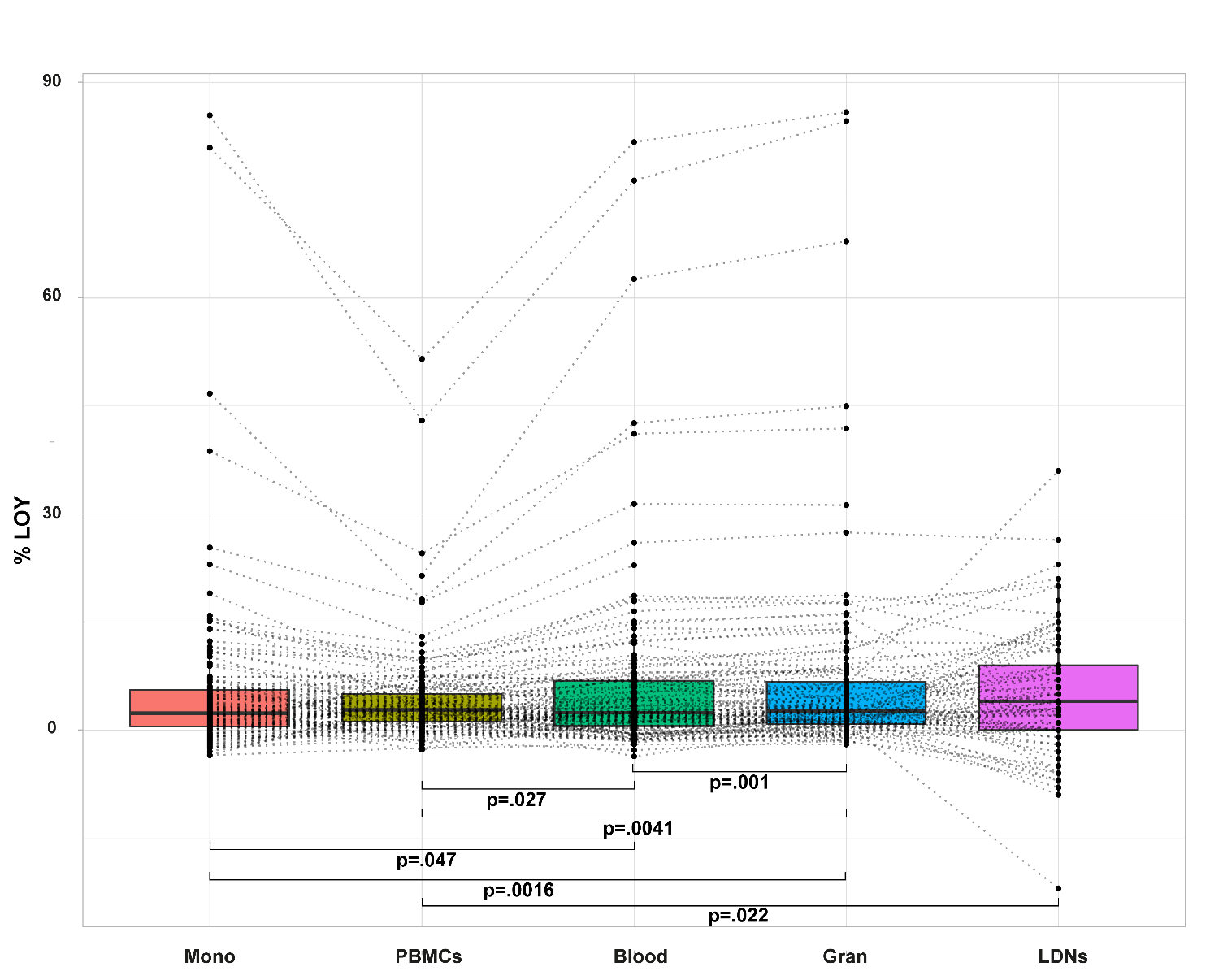


**Fig. S3. Comparison of %LOY across five cell populations for patients during ICU-treatment**. Comparison of unadjusted percentages of loss of chromosome Y (%LOY) in monocytes (Mono, n=132), PBMCs (n=139), whole blood (Blood, n=138), granulocytes (Gran, n=132) and low-density neutrophils (LDNs, n=89) within ICU-patients. Each dotted line connects the data points within the same patient. Boxplots show median, IQR as hinges and largest values no further than 1.5*IQR away as whiskers. Unadjusted p-values ≤0.05 from paired two-sided Mann-Whitney-Wilcoxon non-parametric tests are shown.
